# Gene Expression in Response to Exercise in Patients with Chronic Fatigue Syndrome: A Pilot Study

**DOI:** 10.3389/fphys.2016.00421

**Published:** 2016-09-22

**Authors:** Andrew Keech, Ute Vollmer-Conna, Benjamin K. Barry, Andrew R. Lloyd

**Affiliations:** ^1^School of Medical Sciences, University of New South WalesSydney, NSW, Australia; ^2^School of Psychiatry, University of New South WalesSydney, NSW, Australia; ^3^Neuroscience Research AustraliaSydney, NSW, Australia; ^4^Inflammation and Infection Research Centre, School of Medical Sciences, University of New South WalesSydney, NSW, Australia

**Keywords:** myalgic encephalomyelitis, post-exertional malaise, central sensitisation, mRNA, pathogenesis

## Abstract

Chronic fatigue syndrome (CFS) is a debilitating disorder of unknown pathogenesis, characterized by fatigue, which is exacerbated after minimal exercise. We examined the effect of a single bout of aerobic exercise on leucocyte mRNA expression of genes putatively linked to exaggerated afferent signaling as an under-pinning of the fatigue state. A carefully-characterized sample of patients with CFS (*N* = 10) and healthy matched control participants (*N* = 12) were included. Participant ratings of fatigue and other symptoms, as well as blood samples, were obtained at baseline, and five other time-points up to 72 h after 25 min of moderate-intensity cycling exercise. Leucocyte mRNA of 19 metabolite-sensing, adrenergic, immune, and neurotransmission genes was examined using quantitative polymerase chain reaction. Patients with CFS reported substantial fatigue, functional impairment, and poor sleep at baseline (all *p* < 0.02), and exercise immediately induced worsened patients' fatigue (effect size, ES = 1.17). There were no significant changes in gene expression after exercise and patients did not differ from control participants at any time point. Higher levels of expression of ficolin (FCN1) and a purinergic receptor (P2RX4) in patients with CFS were found when all time points were combined. Patients with CFS did not show significant exercise-induced changes in leucocyte mRNA of 19 metabolite-sensing, adrenergic, immune and neurotransmission genes despite a prominent exacerbation of fatigue.

## Introduction

Chronic fatigue syndrome (CFS) is a complex medical condition characterized by persistent, often debilitating fatigue (Fukuda et al., 1994). The disorder is also characterized by a post-exertional exacerbation of fatigue and symptoms, a phenomenon commonly termed “post-exertional malaise.” The fatigue and related symptoms, as well as functional capacity often take hours to days to return to baseline levels (Light et al., 2012; Keech et al., 2015).

It has been proposed that CFS results from a “sensitization” in neural pathways in the brain leading to inappropriate interpretation of physiological signals, ultimately leading to the conscious sensation of fatigue. These physiological signals may originate in the periphery, or from within the brain (Nijs et al., 2012). Recent investigations utilizing physical exercise to analyse correlates of the post-exertional exacerbation phenomenon have raised the possibility of a disturbance in afferent signaling originating in skeletal muscle during exercise (Light et al., 2012; Meyer et al., 2013). Light et al. reported increased leucocyte gene expression after moderate-intensity exercise in patients with CFS but not in matched healthy control participants, specifically for sensory (metabolite-detecting) receptors (e.g., ASIC3, P2RX4), adrenergic receptors (e.g., α-2A, B1, B2), and immune (e.g., IL-6, IL-10) genes (Light et al., 2012). These post-exercise changes in gene expression correlated with the exacerbation in self-reported fatigue and pain recorded in the 48 h following the exercise challenge. Some of these sensory, adrenergic, and immune receptors have previously been implicated in sensory neuron signaling of muscle fatigue and pain in the mouse (Light et al., 2008), hence the authors speculated that leucocytes may act as a surrogate for comparable changes within the muscle.

This study investigated leucocyte gene expression, representing functional pathways possibly associated with the sensation of fatigue, in carefully matched participants with CFS and healthy individuals before and after moderate-intensity exercise. It was hypothesized that patients with CFS would display differential induction of leucocyte gene expression when compared to matched control participants at baseline, and display further increases in gene expression following exercise, correlating with the post-exertional exacerbation of fatigue. By replicating many aspects of the methodology previously applied by Light et al. (2012), especially the analysis of the same genes found to be differentially expressed in patients with CFS following exercise, this study would provide an independent analysis with the added value of repeated baseline measures, and an extended assessment period (up to 72 h following exercise). Additional genes with potential relevance to the sensation of fatigue were also included. These included neuro-behavioral genes, such as those involved in the serotonergic system (SLC6A4, MAOA), which have been hypothesized to be associated with fatigue through its interaction with hypothalamus-pituitary-adrenal (HPA) axis function (Smith et al., 2006). Genes involved in complement activation and the lectin pathway (FCN1, C4a) were also included, due to preliminary evidence of altered expression in patients with CFS following exercise challenge (Sorensen et al., 2009).

## Methods

A well-characterized sample of patients with CFS (*N* = 10) and healthy matched control participants (*N* = 12) were assessed multiple times before and after a moderate-intensity aerobic exercise challenge. A full description of data regarding study participant characteristics, exercise challenge performance, and self-report assessment values has been previously published (Keech et al., 2015). Patients were drawn from a specialist tertiary care clinic for management of the disorder, having been diagnosed by a specialist according to the international criteria (Fukuda et al., 1994). As such, all patients had a relatively stable pattern of symptoms and well managed mood and sleep-wake cycle, and were deemed by their treating clinician to be able to meet the physical demands of the exercise challenge. Patients were excluded if taking medications that influence hypothalamic-pituitary axis function, autonomic nervous system function, or cytokine levels (e.g., beta-blockers), or had a contraindication to participation. Healthy control participants were recruited from university staff and students by advertisement. Patients and control groups were matched by age, sex, BMI, and self-reported levels of physical activity (typical hours per week of at least moderate intensity exercise). The study was approved by the institutional Human Research Ethics Committee. All participants provided informed, written consent.

The exercise challenge consisted of 25 min of moderate-intensity (70% of age-predicted maximum heart rate; APMHR) cycling. APMHR was determined using a standard formula [208 − (0.7 ^*^ age)] (Tanaka et al., 2001). Participants rode on a cycle ergometer (Monark 828E, Sweden) with cadence set at 50 revolutions per minute. The protocol started at 50 watts and workload increased incrementally during the first 5 min of the session until the target heart rate was reached. Workload was regularly adjusted to maintain a heart-rate range of ±3 beats/min of the target. Participants wore a heart rate monitor and a mask connected to a metabolic cart (open-circuit indirect calorimetry system; Medgraphics Ultima CPX, Minnesota, USA) to allow for continuous measurement of heart rate and gas exchange values. RPE was obtained using the numeric, unmodified (6–20) Borg scale (Borg, 1982), and was recorded every 5 min during the session.

Baseline assessments were conducted 24 h before the challenge and again immediately prior to the challenge. Post-exercise assessments were conducted immediately following challenge (post-0) and again at 1, 4, 24, and 72 h after the exercise bout. All exercise challenges were conducted in the morning. Participants attended the lab for the exercise session and the assessments conducted on that day; all other assessments were performed at the participant's home to minimize the effect of travel-induced fatigue in the patients. Participants rested in the lab for the first hour following the exercise session, and were asked to maintain normal daily routine for the entirety of the assessment period (plus the 24 h leading in to the initial assessment). Participants were also asked to abstain from caffeine or alcohol consumption for 4 h prior to the assessments and in the lead-up to sleep each night.

Participants were assessed for measures of gene expression in association with the self-report of fatigue, using the Fatigue and Energy Scale (FES; Keech et al., 2015). Physical symptoms, sleep quality, and the level of functional impairment were obtained at the initial baseline assessment via the SOMA sub-scale of the Somatic and Psychological HEalth REport (SPHERE), the Pittsburgh Sleep Quality Index (PSQI), and the Brief Disability Questionnaire (BDQ), respectively (Buysse et al., 1989; Von Korff et al., 1996; Hickie et al., 2001).

### Gene expression

Blood samples were collected in acid-citrate dextran (ACD) tubes and processed within 4 h of sampling. Peripheral blood mononuclear cells (PBMCs) was separated (Lymphoprep; AXIS-SHIELD, Norway), and then lysed in Tri Reagent (Sigma, USA) and immediately stored at −80°C. RNA was extracted using standard procedures (i.e., PBMCs were thawed and resuspended, cells lysed with Trizol and separated using chloroform, the extracted RNA was precipitated with isopropanol, washed with ethanol and resuspended in DEPC water). RNA integrity was confirmed by Agilent 2100 bioanalyser (Agilent Technologies, Germany) with a mean RNA integrity number (RIN) of 9.4 (range 8.2–10). cDNA libraries were synthesized using the ABI High Capacity cDNA Archive kit (Applied Biosystems, Inc., USA) and stored at −20°C until analysis. Samples were analyzed using a quantitative, real-time PCR system (7900HT, Applied Biosystems, Inc.) using ABI TaqMan Master Mix (Applied Biosystems, Inc.). Master mix and primer probe solutions and template solutions were separately loaded onto 96-well plates before centrifugation to remove any air bubbles. Each sample was run in duplicate with standards run in quadruplicate. Primer probes (all from TaqMan Gene Expression Assays; Applied Biosystems, Inc.) were as follows: ASIC3 (Hs00245097_m1); P2RX4 (Hs00602442_m1); P2RX5 (Hs00531938_m1); P2RX7 (Hs00175721_m1); TRPV1 (Hs00218912_m1); adrenergic α-2A (Hs00265081_s1); adrenergic β-1 (Hs02330048_s1); adrenergic β-2 (Hs00240532_s1); COMT (Hs00241349_m1); IL-6 (Hs00985639_m1); IL-10 (Hs00961622_m1); IL-1β (Hs01555410_ m1); IFN-γ (Hs00989291_m1); FCN1 (Hs00157572_m1); MAOA (Hs00165140_m1); MASP2 (Hs00198244_m1); NPY (Hs00173470_m1); TLR4 (Hs01060206_m1); CD14 (Hs00169122_g1); PRF1 (Hs00169473_m1); SLC6A4 (Hs00169010_m1); LTA (TNF-α superfamily) (Hs00236874_m1); C4a/C4b (Hs00246758_m1) and the control probe 18-S (Hs99999901_s1).

Real-time PCR results were analyzed with SDS 2.2 Software and Data Assist v.3 (both Applied Biosystems, Inc.) and inspected for errors in processing (e.g., loading errors, robot errors, threshold errors). Data were analyzed according to the ddC_T_ method described in ABI User Bulletin#2 (Applied Biosystems, Inc.). The maximum acceptable C_T_ (threshold cycle) value was set at 38. Genes were excluded from analysis if C_T_ values were greater than 38 for greater than 30% of all samples; this was the case for three genes (NPY; MASP2; adrenergic α-2A). The maximum C_T_ value (i.e., 38) was included in calculations for all other data points. Within participant datasets and their replicates, outlier values were determined as those at least 2.5 *SD* outside the group mean and at least four-fold different from values obtained at adjacent time-points for that participant. These criteria identified 13 outlier ddC_T_ values (out of 2375 total values); these were replaced by the cut-off criteria (group mean ± 2.5 *SD*). Other missing values (7 out of 2375 total values) were replaced by the group mean for the difference between adjacent time-points added to the individual's previous time-point value.

### Statistical analysis

All analyses were performed using SPSS v20.0 (SPSS Inc., Chicago, IL). Statistical analysis of gene expression involved analysis of ddC_T_ values for each gene (relative to the reference gene) at each assessment-point. In this process, ddC_T_ values were log (10) transformed to allow for parametric statistical analysis. The baseline value of measures of gene expression was taken as the mean of two pre-exercise assessments. Post-exercise values for each gene expression measure were normalized relative to the same participant's baseline levels, allowing for analysis of fold change. For two genes (IL-1β and PRF1), the log transformation created a combination of negative and positive values, which would not allow for analysis of fold change; in both instances, a constant value of 1 was added to all raw ddC_T_ values for each gene.

Data were tested for normality using the Shapiro-Wilkes Test. Independent *t*-tests were used to assess for group differences in participant characteristics, self-reported fatigue and symptom measures, baseline levels of gene expression (using the ddC_T_ values), and exercise challenge performance; a Chi-square test assessed sex distribution. Non-normally distributed data were analyzed using the Mann-Whitney test. Exploratory analysis of gene expression and fatigue measures was conducted via 2-way repeated measures ANOVA (2 groups × 6 time-points), and a multivariate repeated-measures MANOVA was applied to analyse across gene categories (metabolic-sensing; adrenergic; immune; neurotransmission). Paired samples *t*-tests were applied as follow-up tests to assess within group changes for all repeated measures compared to baseline, and effect sizes were calculated using Cohen's *d*. Group comparisons of post-exercise gene expression were also run on the mean fold change from baseline for all post-exercise time-points combined, and correlation analyses (Pearson's *r*) sought two-tailed bivariate associations between this measure for each gene and the severity of self-reported fatigue after exercise (Area Under Curve, as determined from sum of change from baseline values for all post-exercise time-points combined). Significance for statistical measures involving gene expression data was set at *p* < 0.01, given the number of gene expression parameters analyzed (i.e., 19 genes per time-point). Data in text and tables are presented as mean ± standard deviation (*SD*); figures are presented as mean ± standard error of the mean (SEM).

In the gene expression dataset, the data from the control probe, 18-S (Hs99999901_s1) showed an unsatisfactorily high coefficient of variance (CV) across samples (7.4%) and a significant difference in cycle counts (C_T_ values) between groups (*p* < 0.001). Accordingly, CD14 was chosen as the reference gene based on the lack of significant variation within-participants over time (CV 3.4%), and closely comparable CT values between groups (25.7 ± 0.9 for control participants and 25.6 ± 0.9 for patients; *p* = 0.66). This gene has similarly been reported to be invariant in patients with CFS, including after exercise (Light et al., 2012; Meyer et al., 2013). All gene expression levels were normalized relative to this control probe.

## Results

In brief, the study involved patients with CFS (*N* = 10, 6 female; 41.4 ± 8.4 years) and healthy control participants (CON: *N* = 12, 8 female; 34.1 ± 10.2 years) of comparable body mass index (BMI) (CFS: 22.1 ± 3.2; CON: 24.1 ± 1.9; *p* = 0.09) and physical activity levels (CFS: 1.2 ± 1.0 h/week; CON: 1.7 ± 1.4 h/week; *p* = 0.39). Consistent with the diagnosis of CFS, patients reported higher baseline levels of fatigue (FES) (CFS: 5.1 ± 1.1; CON: 0.4 ± 0.7; *p* < 0.001), more physical symptoms (SOMA) (CFS: 6.6 ± 2.4; CON: 0.7 ± 1.1; *p* < 0.001), lower quality of night-time sleep (PSQI) (CFS: 7.6 ± 2.2; CON: 2.9 ± 2.1; *p* < 0.001) and less physical function (BDQ) (CFS: 13.1 ± 4.4; CON: 0.2 ± 0.4; *p* < 0.001) in the baseline assessment than control participants. No participant reported performing any strenuous exercise or cognitive activity in the 24 h leading in to the initial baseline assessment, or during the entire assessment period (other than the exercise challenge). All participants completed the full duration of the exercise challenge without any breaks and there were no significant between-group differences in most indices of exercise performance, however patients consistently reported higher ratings of perceived exertion (RPE) (mean values drawn from the last 20 min of exercise: CFS: 15.3 ± 1.8; CON: 11.5 ± 1.3; *p* < 0.001).

In response to exercise, patients with CFS reported higher levels of fatigue than the CON group at each assessment-point after exercise (all *p* < 0.001). In control participants, mean ratings of fatigue on the FES over the assessment period never exceeded 1 (described on the scale as *just noticeable*) out of 10, and exercise did not induce any significant increase in fatigue compared to baseline. By contrast in patients with CFS, ratings of fatigue increased significantly immediately following exercise (7.1 ± 1.6; ES = 1.53) and raised levels of fatigue were maintained for 24 h after the trial (6.6 ± 1.9) (both *p* ≤ 0.025). These scores reflect a change in mean fatigue severity from *moderate* levels before exercise to *high* levels after exercise.

### Gene expression

**Figure 1:**
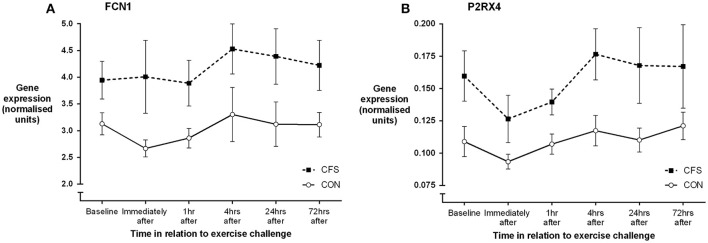
**Mean (SEM) gene expression (ddC_**T**_, relative to the reference gene) for (A) FCN1 and (B) P2RX4 before and following moderate intensity aerobic exercise in patients with CFS (***N*** = 10) and matched healthy control participants (***N*** = 11)**.

**Figure 2:**
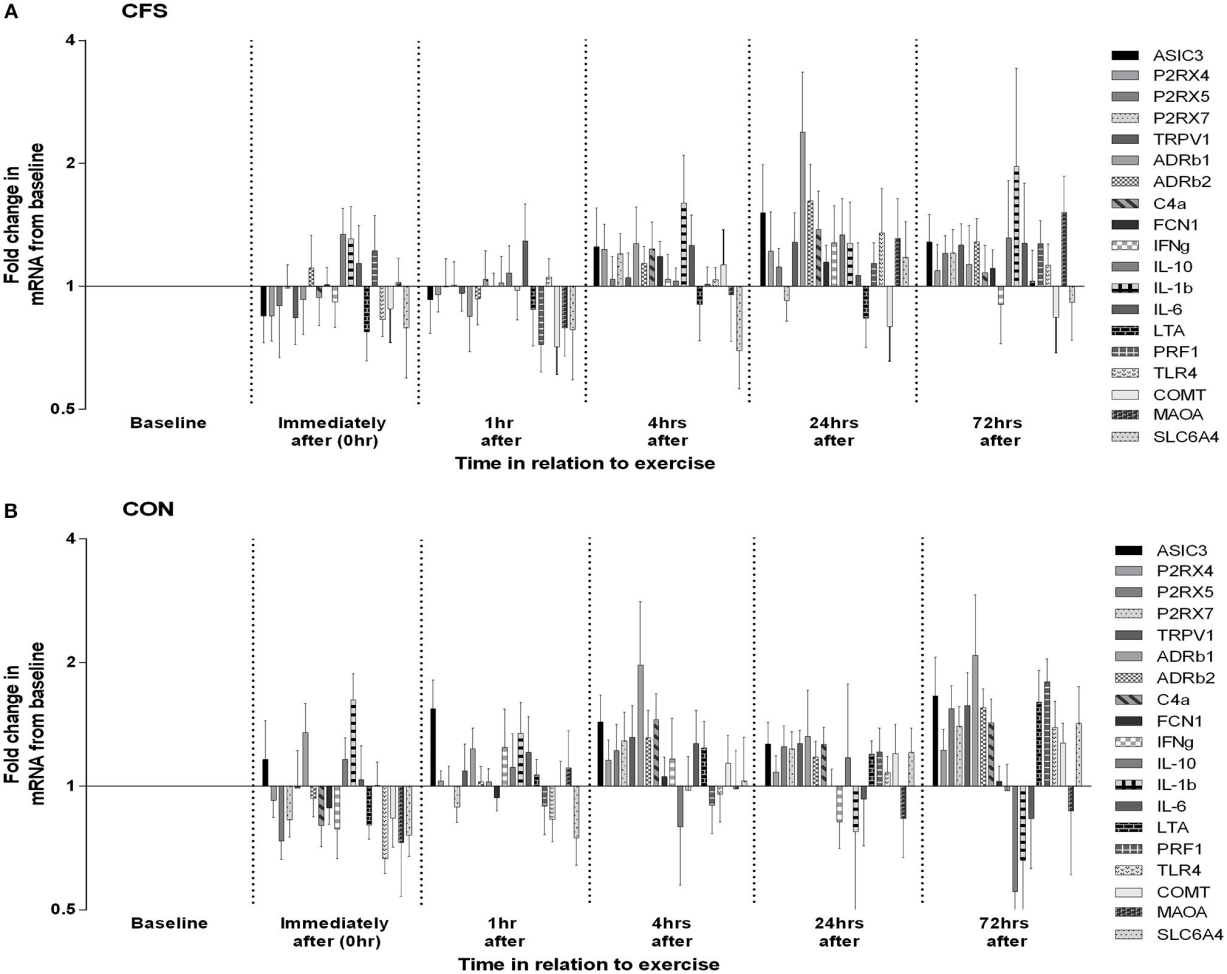
**Changes in leucocyte gene expression following moderate intensity aerobic exercise in (A) patients with CFS (***N*** = 10) and (B) matched healthy control participants (***N*** = 11)**. Data for each gene at each time-point are depicted as mean (SEM) fold increases from pre-exercise baseline. Graphs are plotted on log_2_ scale.

One control participant did not provide blood samples (CON: *N* = 11). No significant between-group differences in mRNA levels (ddC_T_ values) were seen at baseline or in the mean post-exercise fold change levels for any of the 19 gene receptors of interest (all *p* > 0.03; Table 1). MANOVA analysis of genes grouped according to functional pathway also showed no significant group differences at baseline or following exercise (all *p* > 0.05; Table 1). There was a main effect between groups across all assessment points (i.e., combining before and after exercise values) for 2 genes: FCN1 (*p* = 0.009) and P2RX4 (*p* = 0.002; Figure 1). There was no significant effect of exercise on any gene for the patient group, or a group × time effect for any gene. Figure 2 shows the mean fold change in ddC_T_ values from baseline for each of the gene receptors for each of the assessment-points after exercise. The range of fold change from baseline for the CFS group was 0.70 (SLC6A4 at post-4 h) to 2.39 (adrenergic β-1 at post-24 h); and for the CON group, 0.55 (IL-10 at post-72 h) to 2.08 (adrenergic β-1 at post-72 h). Baseline levels of fatigue did not correlate with any baseline measure of gene expression, and post-exercise change in fatigue levels did not correlate with the post-exercise mean fold change in the level of expression (in relation to baseline) for any gene.

**Table 1:** **Means (±***SD***), ***t***-test, effect size and MANOVA results for baseline ddCT values for mRNAs relative to the reference gene, and post-exercise expression fold change values relative to baseline, from patients with CFS (***N*** = 10) and control participants (***N*** = 11)**.

**Gene receptor**	**Baseline values**				**Post-exercise values**			
	**CFS**	**Controls**	***p***	***d***	**CFS**	**Controls**	***p***	***d***
**Metabolite-detecting**		**MANOVA**	**0.11**	**0.66**		**MANOVA**	**0.52**	−**0.23**
ASIC3	0.0090 ± 0.0079	0.0047 ± 0.0019	0.17	0.75	1.16 ± 0.66	1.41 ± 0.8	0.42	−0.34
P2RX4	0.1597 ± 0.0614	0.1090 ± 0.0385	0.03	0.99	1.07 ± 0.35	1.07 ± 0.28	0.99	0
P2RX5	0.2149 ± 0.1559	0.195 ± 0.1226	0.71	0.14	1.05 ± 0.36	1.15 ± 0.38	0.55	−0.27
P2RX7	0.0416 ± 0.0169	0.0302 ± 0.0109	0.08	0.80	1.07 ± 0.32	1.13 ± 0.28	0.64	−0.20
TRPV1	0.0099 ± 0.0054	0.007 ± 0.0033	0.16	0.65	1.08 ± 0.34	1.25 ± 0.58	0.76	−0.35
**Adrenergic**		**MANOVA**	**0.21**	**0.55**		**MANOVA**	**0.33**	−**0.26**
β-1	0.0053 ± 0.0046	0.0033 ± 0.0022	0.22	0.55	1.11 ± 0.61	1.59 ± 0.99	0.31	−0.58
β-2	0.2343 ± 0.1427	0.1724 ± 0.0693	0.22	0.55	1.22 ± 0.33	1.2 ± 0.23	0.86	0.07
**Immune**		**MANOVA**	**0.56**	**0.53**		**MANOVA**	**0.18**	**0.07**
C4a/C4b	0.0093 ± 0.0057	0.0062 ± 0.0025	0.23	0.70	1.14 ± 0.46	1.19 ± 0.33	0.74	−0.12
FCN1	3.9453 ± 1.1122	3.1295 ± 0.6846	0.06	0.88	1.09 ± 0.19	0.98 ± 0.22	0.26	0.54
IFN-γ	0.0182 ± 0.0199	0.0103 ± 0.0066	0.51	0.53	1.02 ± 0.41	1.0 ± 0.44	0.92	0.04
IL-6	0.0071 ± 0.009	0.0051 ± 0.0037	0.61	0.29	1.21 ± 0.58	1.06 ± 0.52	0.54	0.27
IL-10	0.0106 ± 0.0096	0.0086 ± 0.0062	0.99	0.25	1.23 ± 0.37	0.96 ± 0.55	0.21	0.58
IL-1β	0.3893 ± 0.332	0.2851 ± 0.2585	0.31	0.35	1.12 ± 0.49	1.08 ± 0.5	0.81	0.08
LTA	0.0242 ± 0.0243	0.0128 ± 0.0073	0.28	0.64	0.88 ± 0.39	1.18 ± 0.29	0.06	−0.87
PRF1	0.5247 ± 0.298	0.3817 ± 0.1923	0.22	0.57	1.08 ± 0.31	1.16 ± 0.34	0.61	−0.24
TLR-4	0.0237 ± 0.0081	0.0195 ± 0.0065	0.21	0.57	1.08 ± 0.33	0.98 ± 0.23	0.46	0.35
**Neurotransmission**		**MANOVA**	**0.37**	**0.19**		**MANOVA**	**0.46**	−**0.17**
COMT	0.2918 ± 0.1653	0.1776 ± 0.107	0.04	0.82	0.87 ± 0.43	1.09 ± 0.43	0.26	−0.51
MAOA	0.0013 ± 0.0017	0.0019 ± 0.0016	0.11	−0.36	1.07 ± 0.43	0.91 ± 0.55	0.46	0.32
SLC6A4	0.005 ± 0.0046	0.0045 ± 0.0049	0.76	0.11	0.87 ± 0.53	1.03 ± 0.4	0.44	−0.34

ASIC3, acid-sensing ion channel; P2RX4, purinergic type 2RX4 receptor; P2RX5, purinergic type 2RX5 receptor; P2RX7, purinergic type 2RX7 receptor; TRPV1, transient receptor potential vanilloid type 1; COMT, catechol-O-methyltransferase; C4a/C4b, Complement C4-a/Complement C4b; FCN1, ficolin-1; IFN-γ, interferon-γ; IL-6, interleukin-6; IL-10, interleukin-10; IL-1β, interleukin-1β; LTA, lymphotoxin-α (TNF-β superfamily); PRF1, perforin 1; TLR4, toll-like receptor 4; MAOA, monoamine oxidase-A; SLC6A4, serotonin transporter.

Baseline values were the average of the two assessment time-points prior to exercise. Post-exercise analysis includes the mean change in expression, in relation to baseline levels (= 1.00), across all post-exercise assessment time-points.

## Discussion

This study analyzed gene expression before and after moderate-intensity aerobic exercise in a well-characterized patient group with CFS and healthy control participants. The prominent exacerbation of fatigue following exercise was not accompanied by any significant abnormalities in expression of the metabolite-sensing, adrenergic, immune, or neurotransmission genes in the patient group, either in comparison to healthy control participants or within the patient group following exercise. There was also no difference between the patient group and the control group in baseline levels of gene expression prior to the exercise bout. These findings do not replicate the closely comparable study by Light et al., which noted prominent changes in expression of many of these genes following exercise in patients with CFS (Light et al., 2012). However, the gene expression data are generally consistent with the findings by Meyer et al. who did not observe differential expression between patients and control participants for 12 of the 14 genes studied, with the exception of adrenergic α-2A and NR3C1 (a glucocorticoid receptor), neither of which was studied here (Meyer et al., 2013). Each of these prior studies applied an exercise challenge to induce post-exertional exacerbation of symptoms in patients with CFS, and utilized comparable gene expression testing and statistical analysis procedures.

Despite similar methodologies, there are some key differences between these three studies. Firstly, the Light et al. dataset included a subset of patients with severe functional disability, characterized as being predominantly house- or bed-bound with minimal activity tolerance and dependent upon others for activities of daily living (Light et al., 2012). This subset of patients showed significant increases in gene expression following exercise, in contrast to the relatively mild increases in gene expression seen in the rest of the patient group, who were reporting up to moderate disability. Our study did not include any patients with severe functional disability, and so could not examine the possibility of disordered gene expression following exercise for participants with more severe CFS. Although the patient group reported here are more typical of the illness severity and functional capacity of patients with CFS, further studies to test more severely affected patients are warranted—to consider whether the reported association relates to the disease process itself, or rather reflects secondary pathophysiological changes associated with prolonged illness and inactivity. Secondly, in contrast to the other studies, Meyer et al. applied a maximal aerobic capacity test, which is a more physically intense but shorter duration exercise challenge (Meyer et al., 2013). While it is plausible that the different exercise intensity may have differentially affected gene expression patterns following exercise, the time-course and severity of patients' symptom exacerbation following exercise was similar across all studies. Thirdly, our study differed in the choice of the reference gene chosen for normalization of the gene expression dataset. Light et al. reported that TF2B had less intrinsic variation than other housekeeping candidates, had an expression level that was similar to the genes of interest, and did not vary with the exercise protocol (Light et al., 2012). By contrast, the putative reference gene initially selected in our study (18-S) displayed transcription levels which varied significantly between individuals and were not distributed normally. An alternative reference gene with very limited within-subject and between-subject variation (CD14) was therefore chosen.

The gene expression data did reveal consistently higher levels of expression of ficolin (FCN1) and one of the purinergic receptors (P2RX4) in patients with CFS, but which were not differentially expressed after exercise. These two genes reflect different biological pathways [FCN1—immune (lectin pathway of complement activation); P2RX4—energy metabolism (via ATP)] and there is no clear biological link between the two. With regard to P2RX4, Light et al. observed raised levels of expression following exercise (as reflected in the AUC values) but not at baseline (Light et al., 2012), while Meyer et al. observed no abnormal expression in P2RX4 in patients at baseline or after exercise (Meyer et al., 2013). Neither previously published gene expression study measured ficolin, however an earlier pilot study by Sorensen et al. reported raised levels (>two-fold) of ficolin and C4a expression in at least 4 out of 8 patients with CFS at an assessment time-point 1 h following exercise (Sorensen et al., 2009). It should be noted that Sorensen et al. found that ficolin was not differentially expressed at baseline or 6 h following exercise, and the data reported here did not reveal any abnormality in patients' C4a expression. It should be noted that as previous reports indicate FCN levels vary in monocytic sub-populations (Frankenberger et al., 2008), the choice of CD14 as a normalization transcript may have influenced this analysis.

Of these two genes, P2RX4 is the most likely candidate for further investigation in relation to the pathophysiology of CFS and the “central sensitization” paradigm. P2RX4 has recently been implicated as a key factor in driving neuropathic pain, with raised levels of *de novo* expression within the CNS signifying a specific underlying microglia response phenotype which is critical for the pathogenesis of pain hypersensitivity caused by injury to peripheral nerves (Beggs et al., 2012; Tsuda et al., 2013). A recent preliminary finding of neuro-inflammation, detected using positron emission tomography (PET), in patients with CFS may likewise suggest microglial involvement in the pathophysiology (Nakatomi et al., 2014). In general, ddC_T_ values in the present study reflect low levels of mRNA expression, which varied very little within, or between, participants. Accordingly, the biological significance of the gene expression differences associated with CFS, even if detectable statistically, is questionable.

### Strengths and limitations

This study analyzed a well-characterized patient group with stable mood and optimized sleep-wake cycle patterns, and a control group matched for physical activity levels, and applied two baseline measures and multiple repeated measures post-exercise for each participant. However, a relatively small sample size and the absence of patients on the more severe end of the CFS spectrum limits the ability to draw more definitive conclusions, while the ability to compare these findings with those previously reported by Light et al. and Meyer et al. (Light et al., 2012; Meyer et al., 2013) is limited by the application of a different reference gene. In addition, while expression of genes on leucocytes may plausibly be used as a surrogate of activity of the same genes within the nervous system, there are no published data directly supporting this assertion. Therefore, interpretation of leucocyte gene expression data with respect to nervous system activity should be performed cautiously.

## Conclusion

A bout of moderate-intensity aerobic exercise induced a sustained exacerbation of fatigue in patients with CFS, but was not accompanied by corresponding changes in leucocyte gene expression for a selection of genes involved in metabolite-sensing, adrenergic, immune, or neurotransmission pathways.

## Author contributions

AK designed the study, collected, analyzed and interpreted the study data, and wrote the manuscript. UV, BB, and AL interpreted the study data and contributed to the manuscript. AL secured funding for the project. All authors have read and approved the final manuscript.

### Conflict of interest statement

The authors declare that the research was conducted in the absence of any commercial or financial relationships that could be construed as a potential conflict of interest.
